# Laryngomalacia surgery: a series from a tertiary pediatric hospital

**DOI:** 10.5935/1808-8694.20120041

**Published:** 2015-10-20

**Authors:** José Faibes Lubianca Netto, Renata Loss Drummond, Luciana Pimentel Oppermann, Fernando Stahl Hermes, Rita Carolina Pozzer Krumenauer

**Affiliations:** a PhD in Medicine and Medical Sciences, Federal University of Rio Grande do Sul (Associate Professor of ENT at the Federal University of Health Sciences of Porto Alegre. Head of the Pediatric ENT Service at HCSA. Chairman of the Brazilian Associaton of Pediatric Otorhinolaryngology); b MD (Resident Physician of the ENT Service at the Santa Casa de Porto Alegre); c MD, ENT (MD, ENT); d MD, ENT (MD, ENT); MSc in Health Sciences/Pediatrics (Advisor of the Pediatric ENT Service at the Santa Casa de Porto Alegre - Santo Antônio Children's Hospital)

**Keywords:** laryngomalacia, respiratory insufficiency, respiratory sounds

## Abstract

Laryngomalacia is the condition responsible for 75% of the cases of stridor in children aged up to 30 months, in which there is supraglottic collapse during inhalation. Inspiratory stridor is a characteristic telltale. As many as 20% of the patients are severely affected and require surgery. Supraglottoplasty is the procedure of choice and the presence of comorbidities is the most relevant prognostic factor for surgery success.

**Objective:**

To describe a series in a tertiary pediatric hospital, its success rates, and surgery prognostic factors.

**Method:**

This retrospective cohort study enrolled 20 patients submitted to supraglottoplasty between July 2007 and May 2011.

**Results:**

Thirteen (65%) patients were males; mean age at the time of the procedure was 6.32 months. Endoscopic examination showed that 12 subjects had combined forms of laryngomalacia, 40% had associated pharyngomalacia, and three also had tracheomalacia. Thirteen subjects had isolated laryngomalacia and seven had gastroesophageal reflux disease. Fifteen (75%) patients underwent aryepiglottic fold resection. After the procedure, eleven patients were asymptomatic and two required tracheostomy. Presence of comorbidities was the strongest predictor of unfavorable postoperative outcome (*p* = 0.034).

**Conclusion:**

Supraglottoplasty is a safe therapeutical procedure for select patients with laryngomalacia.

## INTRODUCTION

Laryngomalacia is a condition in which supraglottic tissues collapse in a cycle during inhalation to produce mild, moderate, or severe respiratory obstruction. It is the most common cause of stridor in children and accounts for 60% to 75% of the cases in children aged 30 months or less^1^. The real incidence rate of laryngomalacia is unknown and severe cases are predominantly diagnosed at tertiary pediatric hospitals^2^. The pathophysiology of this condition is still uncertain. Initially, laryngomalacia was thought to occur as a consequence of the inability of immature cartilages to offer a stiff framework for the larynx, but this explanation has been refuted by histopathology tests in which cartilage alterations were not observed in laryngomalacia patients^3^. Today, the most widely accepted theory suggests that laryngomalacia is the outcome of disordered laryngeal neuromuscular tone^4^, ^5^. This idea is supported by biopsy studies that revealed histopatholo-gical alterations in laryngeal nerve tissue of children with severe laryngomalacia^6^.

The characteristic clinical finding is inspiratory stridor occurring between the second and sixth week of life of the subject, which tends to resolve spontaneously before the individual reaches the age of two^1^. As many as 20% of the patients, depending on the level of care complexity given at the facility the patient is hospitalized, have severe laryngomalacia characterized by upper airway obstruction, acute life-threatening events, low weight gain, and slow growth. These patients are the best candidates for surgical treatment^7^.

Supraglottoplasty was originally described in 1922^8^, and became the procedure of choice to treat laryngomalacia. For a long time, tracheostomy was the only treatment available to avoid fatal events, but it has been increasingly less offered to patients. Many approaches have been described, and success rates range between 38.1% and 100%^9^. Apparently, the most relevant factor in the failure of surgical treatment is the presence of comorbidities, a finding observed in about 45% of the patients (neurological disease, cardiopathy, genetic syndrome or anomaly)^10^, ^11^. Additionally, some 30% of the pediatric patients may have synchronous airway injuries^2^, ^3^, ^4^, ^5^, ^6^, ^7^, ^8^, ^9^, ^10^, ^11^, ^12^, ^13^, ^14^ and 85% to 96% of these patients may have laryngeal edema or pharyngolaryngeal reflux^15^.

Current procedures are based on individualized care and the anatomic and functional alterations observed in each patient. Thus, when the issue is shortened aryepiglottic folds, resection with microscissors^2^, laser^16^, or microdebrider^17^ is recommended. If there is associated redundant arytenoid mucosa or cuneiform cartilages, supraglottoplasty - a more generic term that encompasses the resection of the aryepiglottic folds, excess mucosa, and the lateral surface of the epiglottis^18^ - is recommended. If the determining factor for the observed obstruction is epiglottis inhalation, partial epiglottectomy or glossoepiglottopexy^19^ is recommended.

This paper aimed to describe a patient series seen at a Brazilian tertiary pediatric hospital, surgery success rates, and analysis of prognostic factors.

## METHOD

This is a prospective cross-sectional study on a historical cohort based on patient chart reviews. Patients with severe laryngomalacia submitted to endoscopic surgery between July 2007 and May 2011 at a tertiary pediatric ENT service in Brazil were enrolled in the study. Twenty patients were included. This study was approved by the Research Ethics Committee and given permit 3682/11.

The following clinical variables were observed in the diagnosis of laryngomalacia: onset of stridor, factors connected to improvements and decays in subject overall condition, perinatal history, presence of other congenital anomalies, difficult breastfeeding, weight gain, cyanosis episodes, and presence of apnea and other events requiring hospitalization for respiratory obstruction.

Patients suspected for laryngomalacia underwent nasal bronchoscopy in a surgical center on a Machida® 3.2 mm endoscope. Subjects were offered inhalation anesthesia and spontaneous ventilation support, and had their airways monitored to the level of the secondary bronchi. Patient examinations were recorded on a DVD. Laryngomalacia was classified into five types, according to the scheme proposed by Holinger^20^: type I (prolapse of enlarged cuneiform cartilages), type II (omega-shaped epiglottis that folds on itself during inhalation), type III (anterior and medial collapse of the arytenoids during inhalation) and type V (shortened aryepiglottic folds); patients may have more than one type of alteration. Rigid bronchoscopes were not used in the assessment of the patients included in this study.

The criteria for severe laryngomalacia with surgical indication were as follows: a) clinical evidence of severe respiratory obstruction such as cyanotic episodes with or without supracostal and intercostal retractions and/or pectus excavatum and/or b) swallowing disorders accompanied by low weight gain and deficient stature gain.

Patient charts were reviewed for history of diseases and particular emphasis was given to comorbidities such as GERD, neurological disease, and cardiopathy as per the protocol defined before data collection. (Annex 1).

**Annex 1:** Pediatric ent service post-supraglottoplasty protocol.

Name: _____________________________________________________________________
IH: _________________________________ HMO: _________________________________
Phone:
Patient age at the time of the procedure:
Procedure date:
Type of laryngomalacia:
() I (prolapse of enlarged cuneiform cartilages)
() II (omega-shaped epiglottis that folds on itself during inhalation)
() III (anterior and medial collapse of the arytenoids during inhalation)
() IV (posterior projection of the epiglottis during inhalation)
() V (shortened aryepiglottic fold)
Associated pharyngomalacia:
() Yes
() No
Type of procedure:
() Aryepiglottic fold resection
() Resection of posterior redundant tissue
() Glossoepiglottopexy
() Unilateral
() Bilateral
Complications during the procedure:
() Não
() Yes. Describe: ________________________
Length of ICU stay: ___ hours
Length of postoperative hospitalization: __ days
Immediate preoperative care:
() OTI
() no OTI
Intraoperative:
() OTI
() no OTI
Immediate postoperative care:
() no OTI
() OTI if so: __________
Time of extubation in the ICU (postoperative care): _____ days
Comorbidities:
() Cardiopathy: _______________________
() Neuropathy ________________________
() Craniofacial deformities: _____________________________
() Down S. () Preterm birth
() GERD
() Other: ___________________________
NPL: Date:
Report:
Comments:

GERD was considered present in patients with clinical signs of reflux (vomiting, low weight gain, abdominal discomfort), as in most cases the children got to our service after being referred by other physicians with an established diagnosis and/or already in treatment for GERD. Additionally, in our institution pH measurement is not offered to all individuals assessed for GERD, as it requires hospitalization.

### Surgical Approach and Follow-up

In 14 patients, surgery was done with inhalation (sevoflurane) and intravenous (propofol and fentanyl) anesthesia, without orotracheal intubation. One of the remaining six underwent a tracheostomy and the other five were intubated because of comorbidities or difficulty maintaining the proper anesthetic plane during the procedure.

The larynx was exposed through suspension direct laryngoscopy (Parsons laryngoscope) adapted to the size of the patient. An oxygen line was positioned in the supraglottis close to the vocal folds under direct visualization. Primary hemostasis was done with the aid of a microscope, and cotton balls soaked in adrenalin 1:9000 and tied to a silk string were placed on the aryepiglottic folds and arytenoid cartilages. Then the arytenoid folds were incised bilaterally and the redundant soft supraglottic tissues were resected with Karl Storz pediatric microlaryngoscopy microforceps and microscissors (cold instruments). Glossoepiglottopexy was performed in one case with a monopolar cautery to revitalize the base of the tongue and the lingual face of the epiglottis, and produce synechia. Hemostasis was then carefully revised, suction applied to residual bleeding, Table 1 continued and adrenalin-soaked cotton balls placed again when needed, before they were removed at the end of microlaryngoscopy.

**Table 1:** Demographics.

	Gender	Age	CX	Type of Laryngomalacia	Procedure	Comorbidity	GERD
1	M	4	V	N	Aryepiglottic fold resection	Macroglossia	N
2	F	4	II e V	N	Aryepiglottic fold resection	N	N
3	M	2	II e V	N	Aryepiglottic fold resection	N	N
4	M	3	V	faringomalácia	Aryepiglottic fold resection	Pierre-Robin S.	S
5	M	4	II, III e V	faringomalácia	Aryepiglottic fold resection	N	N
6	M	6	II, IV e V	N	Aryepiglottic fold resection	N	N
7	F	2	III	N	Aryepiglottic fold resection	N	S
8	F	4	V	traqueomalácia	Aryepiglottic fold and redundant tissue resection	N	N
9	F	9	II e V	N	Aryepiglottic fold resection	N	N
10	M	5	II e V	faringomalácia	Aryepiglottic fold resection	Preterm birth	S
11	M	31	I e V	faringomalácia	Aryepiglottic fold resection	N	S
12	F	9	II e V	faringomalácia	Aryepiglottic fold resection	Down S.; Preterm birth; PDA; IAC; PH; O2-dependent	N
13	M	3	II e V	N	Aryepiglottic fold resection	N	N
14	M	8	II e V	traqueomalácia	Aryepiglottic fold resection	DNPMD	S
15	M	2	V	N	Aryepiglottic fold and redundant tissue resection	PAS	N
16	F	0.5	IV	faringomalácia	Glossoepiglottopexy	Micrognathism	N
17	M	8	IV e V	N	Aryepiglottic fold resection	N	N
18	M	7	II e III	N	Aryepiglottic fold resection	CHARGE; Pierre-Robin S.; O2-dependent	S
19	F	6	I, III e IV	faringomalácia e traqueo-malácia	Aryepiglottic fold and redundant tissue resection	Leukoencephalopathy; DNPMD	S
20	M	5	V	faringomalácia	Aryepiglottic fold resection	N	N

GERD: gastroesophageal refux disease; PDA: patent ductus arteriosus; IAC: interatrial communication; PH: pulmonary hypertension; DNPMD: delayed neuropsychomotor development; PAS: pulmonary artery stenosis; CHARGE: coloboma, hearing impairment, atresia of the choanae, retardation of growth, genital abnormalities, and cardiopathy; Leukoencephalopathy: multicystic leukoencephalopathy by perinatal anoxia; TCT: tracheostomy.

All patients were sent to the pediatric ICU after surgery. They were extubated whenever possible. Systemic steroids and anti-reflux medication were administered as a routine.

The patients were discharged as soon as their airways were secured, they were on spontaneous ventilation, and supplementary oxygen therapy was no longer needed. Oral steroids in decreasing dosages were kept for a week, along with anti-reflux therapy. Drug therapy was ceased during outpatient follow-up as subjects evolved favorably. Patients were followed up until their symptoms disappeared and they met the criteria for discharge. Subjects evolving unfavorably underwent additional nasal bronchoscopy and individualized finding-based care.

In this series, the diagnosis of gastroesophageal reflux disease was based on patient history. Anti-reflux therapy was started without pH measurement in all but one case, for whom pH testing had been performed previously as indicated by the then attending pediatrician.

### Statistical analysis

Data analysis was done using statistical package R version 2.12.2 (R Development Core Team [2010]) and electronic spreadsheet Microsoft Excel 2007. An alpha level of 5% was assumed. Data sets were analyzed through descriptive tests and with the aid of a logistic regression model (Agresti [2007]) to assess the impact of variables age, type of laryngomalacia, presence of synchronous airway injury, presence of reflux, and presence of comorbidities upon the supraglottoplasty success rates. In order to statistically represent the size of our sample, outcomes ‘complete symptom resolution' and ‘residual occasional mild stridor' were grouped as favorable outcomes and compared to unfavorable outcomes represented by residual severe symptoms and/or need for tracheostomy. We opted not to assess the type of surgery as a prognostic factor due to the small variance seen on the type of procedure offered to the patients.

## RESULTS

Twenty patients submitted to supraglottoplasty from July 2007 to May 2011 to treat severe laryngomalacia were included in this study. Thirteen (65%) were males. Patient age at the time of the procedure ranged from 12 days to 31 months, and subject mean age was 6.32 months (SD + 6.473 months) (Table 1).

Seven (35%) of the studied patients had neurological, cardiac, craniofacial comorbidities or associated genetic syndromes. Among the cases with comorbidities, one (5%) had Pierre-Robin sequence (PRS), one was a preterm baby, one had impaired neuropsychomotor development, one had Down syndrome (along with patent ductus arteriosus, interatrial communication, and pulmonary hypertension), one had PRS and CHARGE syndrome, and one had impaired neuropsychomotor development and cystic leukoencephalopathy by perinatal anoxia. GERD was seen in six children with the above-mentioned comorbidities. Only one patient had GERD alone (Table 1).

Endoscopic examination revealed associations between different types of laryngomalacia (60%); the most common among isolated types was V (30%). Eight patients (40%) had pharyngomalacia associated to laryngomalacia, and three (15%) had tracheomalacia (Table 1).

Considering the type of surgery, 15 patients (75%) underwent bilateral resection of the aryepiglottic folds; four (20%) also had redundant mucosa resected; and one (5%) had glossoepiglottopexy alone (Table 1).

After surgery, 11 subjects (55%) were symptom-free, five (25%) had mild occasional stridor, and two (10%) had severe residual symptoms and required a tracheostomy. These two patients died in late postoperative care of pulmonary complications (Table 2).

**Table 2:** Outcomes and comorbidities.

Outcome		Comorbidities
Asymptomatic	55% (11)	8 without associated comorbidities
		1 Pierre-Robin S.
		1 micrognathism
		1 CHARGE, Pierre-Robin S.,
		O2-dependent
Mild occasional stridor	25% (5)	2 no comorbidities
		1 macroglossia
		1 preterm birth
		1RDNPM
Persistence of severe symptoms or need for TCT	10% (2)	1 S. Down; Preterm birth; PDA, IAC, PH; TCT; O2-dependent
		1 CHARGE; Pierre-Robin S.; O2-dependent
Lost during follow-up	10% (2)	

PDA: patent ductus arteriosus; IAC: interatrial communication; PH: pulmonary hypertension; DNPMD: delayed neuropsychomotor development; PAS: pulmonary artery stenosis; CHARGE: coloboma, hearing impairment, atresia of the choanae, retardation of growth, genital abnormalities, and cardiopathy; Leukoencephalopathy: multicystic leukoencephalopathy by perinatal anoxia; TCT: tracheostomy.

Mean postoperative follow-up was 5.66 months (SD + 4.82), and ranged from 15 days to 17 months. Two patients (10%) were lost during follow-up as they never returned for their postoperative appointments.

The only variable with statistically significant impact upon the outcome of supraglottoplasty among the ones analyzed (age, type of laryngomalacia, synchronous airway injury, presence of GERD, and presence of comorbidities) was presence of associated comorbidities (*p* < 0.05; Table 3). Absence of comorbidities was associated with a reduction of 48.7% in the probability of patients having severe residual symptoms and/or needing tracheostomy after supraglottoplasty when compared to subjects with comorbidities.

**Table 3:** Logistic regression of post-supraglottoplasty outcome as a dependent variable.

	Estimated coeffcient(β)	Standard error	*p*	Confdence interval
Age	0.092	0.058	0.137	0.021-0.253
Associated airway injury	-0.500	0.290	0.104	-0.476-0.684
GERD	-0.166	0.330	0.621	-0.039-1.281
Comorbidities	-0.666	0.288	0.034*	-0.542-0.610

^*^ *p* < 0.05

## DISCUSSION

This study showed supraglottoplasty was an effective procedure in treating laryngomalacia in select patients. Other authors have alluded to the benefits of this procedure. In 1922, Igauer described the first supraglottic laryngeal procedure to treat laryngomalacia, with partial resection of the epiglottis^8^. Lane et al.^21^, in 1984, excised the lateral portions of the epiglottis and the corniculate cartilages to report significant improvement from laryngomalacia in a three-month-old subject. In 1985, Seid et al.^16^ described the excision of the aryepiglottic folds, and were followed in 1987 by Zalzal et al^19^, who reported on the efficacy of a similar procedure performed in 10 patients. Very few studies in the Brazilian literature have reported on the experience of tertiary hospitals with supraglottoplasty as a treatment for severe laryngomalacia^22^, ^23^, ^24^, ^25^.

Laryngomalacia is the most common cause of congenital stridor. In a previous study, Lubianca et al.^26^ showed that laryngomalacia accounted for 40.8% of the cases of stridor assessed through fiberoptic nasolaryngoscopy and for up to 58% of the cases of stridor in subjects under 30 months. In this study, stridor was the most frequently found symptom leading to the diagnosis of laryngomalacia with all patients being examined through nasopharyngolaryngoscopy. This diagnostic examination proved important in the diagnosis of airway injuries, as such injuries were also correlated with worse postoperative outcome and need for tracheostomy or other symptom alleviation surgical procedures. According to Manusco et al.^14^, approximately 19% of 90 subjects submitted to intraoperative airway assessment had synchronous upper or lower airway injury. In our study, eight subjects (40%) had pharyngomalacia associate with laryngomalacia and three (15%) had associated tracheomalacia.

The indication of supraglottoplasty for children with severe laryngomalacia in this study was based on clinical assessment and fiberoptic nasopharyngolaryngoscopy findings. The clinical criterion used to diagnose severe disease was evidence of severe respiratory obstruction. This has been the assessment used routinely in supraglottoplasty indication. More recently, studies have suggested that polysomnography (PSG) may be used to support the diagnosis of severe laryngomalacia^24^, ^27^, ^28^. However, Avelino et al.^22^ reported that PSG did not add much to clinical follow-up or surgery indication, with parameters low weight gain and pectus excavatum being better predictors for severe laryngomalacia. Up to this moment there is no evidence to support the routine use of PSG in the diagnostic assessment, determination of degree of severity, or surgery indication. None of the patients in our series underwent PSG to support surgery indication.

The surgical approach was individualized as per the fiberoptic nasopharyngolaryngoscopy findings, and the least invasive procedure was picked to solve the main anatomical alterations and avoid the complications described in the literature^9^. It has been shown that most complications are directly related to the extent of surgery, to how much tissue was removed, and to the mode of excision. The division of the aryepiglottic folds and unilateral supraglottoplasty seem to be as effective and less morbid than other more elaborate procedures^9^, ^29^, ^30^. In this study, bilateral resection of the aryepiglottic folds accounted for most of the procedures done on our population. Minor complications such as granuloma, synechia, and aspiration, and major complications such as stenosis, were not observed. Surgery success rate, considering complete symptom resolution and occasional mild stridor as favorable outcomes, reached 80%. This outcome is similar to the one reported by Kelly & Gray^31^, Loke et al.^29^ and Zafereo et al.^27^, who also adopted minimally invasive approaches. None of our patients underwent additional supraglottoplasty procedures to have the excision extended, probably because the excision of both shortened aryepiglottic folds was carried out as a routine.

Subject mean age was 6.32 months (+-6.47). Denoyelle et al.^9^ had 136 supraglottoplasty patients with a mean age of three months. Toyton et al.^32^ described 100 consecutive pediatric patients with severe laryngomalacia submitted to supraglottoplasty, 50% of whom were aged under three months. Hoff et al.^10^ looked into the efficacy of supraglottoplasty and correlated age and associated comorbidities with outcome. They reported that patients with neurological and cardiac comorbidities and associated genetic syndromes were significantly older than subjects without associated comorbidities, in addition to having worse postoperative outcome and need for revision surgery or postoperative tracheostomy. They also observed that patients diagnosed with severe laryngomalacia aged under two months had greater rates of revision surgery. It is believed that cases in which diagnosis was done at a later age were correlated with the presence of other comorbidities such as limited availability of tertiary health care centers, as is the case in Brazil. The study done by Fraga et al.^25^ in Brazil reinforces this idea, as they reported a mean age of diagnosis and surgical indication of 11.5 months.

The presence of associated comorbidities seems to be a determining factor for therapy failure. In our study, seven children (35%) had associated comorbidities, namely craniofacial malformations, genetic syndromes, and neurological and cardiac diseases. Presence of associated comorbidities was the only variable significantly correlated (*p* < 0.05) with unfavorable outcomes, namely persistence of severe symptoms and tracheostomy. According to Senders et al.^33^, presence of comorbidities was correlated with worse short, medium, and long term postoperative outcome. Denoyelle et al.^9^ revealed that when success, complication, recurrence, and supraglottic stenosis rates seen in a group of patients with isolated laryngomalacia were compared to the same rates seen in a group of subjects with laryngomalacia and associated comorbidities, the only variable impacted by the presence of comorbidities was surgery success rate. No differences were reported between the complication and recurrence rates seen in both groups. Hoff et al.^10^ described the presence of cardiac and neurological diseases, and genetic syndromes as determining factors for greater rates of revision surgery and tracheostomy.

Apparently, neurological disorders lead to worse surgery outcome. Fraga et al.^25^ compared children with and without neurological disorders and observed that one half of the subjects with disorder required tracheostomy and the other half had persistent symptoms. Schroeder et al.^12^ also reported a rate of 55% of revision surgery in children with neurological disorders, against 5% in unaffected children. Presence of synchronous airway injury and GERD were not statistically correlated with worse surgery outcome. GERD may have been underdiagnosed, considering that diagnostic pH measurements were not done for all patients. Belmont et al.^11^ and Mathews et al.^15^ suggested that the correlation between reflux and laryngomalacia is causal in some cases and that GERD tends to exacerbate laryngomalacia in others. Hadfield et al.^34^ analyzed reflux through pH measurements before and after aryepiglottoplasty and showed that the resolution of the upper airway obstruction led to less GERD. Data on maintenance of anti-reflux medication during late postoperative care was not adequate for analysis, making it impossible to determine the role of supraglottoplasty in the treatment of gastroesophageal reflux. More efforts are required so that a routine GERD assessment protocol is standardized to determine this condition's actual role in laryngomalacia.

## CONCLUSION

Supraglottoplasty with resection of the aryepiglottic folds is a safe procedure for pediatric patients that allows for significant improvement from severe laryngomalacia in subjects without associated comorbidities.
